# Urban/Rural disparities in Oregon pediatric traumatic brain injury

**DOI:** 10.1186/s40621-015-0063-2

**Published:** 2015-12-11

**Authors:** Megan J. Leonhard, Dagan A. Wright, Rongwei Fu, David P. Lehrfeld, Kathleen F. Carlson

**Affiliations:** 1 Department of Public Health and Preventive Medicine, Oregon Health and Science University, 3181 SW Sam Jackson Park Road, Mail Code CB 669, Portland, OR 97239-3098 USA; 2 Injury and Violence Prevention Section, Oregon Health Authority, 800 NE Oregon Street, Suite 730, Portland, OR 97232 USA; 3 Department of Medical Informatics and Clinical Epidemiology, 3181 S.W. Sam Jackson Park Road, Portland, OR 97239-3098 USA; 4 Department of Emergency Medicine, 3181 SW Sam Jackson Park Road, Portland, OR 97239 USA; 5 Emergency Medical Services & Trauma Systems, Oregon Health Authority, 800 NE Oregon Street, Suite 465, Portland, OR 97232 USA

**Keywords:** Traumatic brain injury, Pediatric, Urban, Rural, Health disparities

## Abstract

**Background:**

Traumatic brain injury (TBI) greatly contributes to morbidity and mortality in the pediatric population. We examined potential urban/rural disparities in mortality amongst Oregon pediatric patients with TBI treated in trauma hospitals.

**Methods:**

We conducted a retrospective study of children ages 0–19 using the Oregon Trauma Registry for years 2009–2012. Geographic location of injury was classified using the National Center for Health Statistics Urban/Rural Classification Scheme. Incidence rates were calculated using Census data for denominators. Associations between urban/rural injury location and mortality were assessed using multivariable logistic regression, controlling for potential confounders. Generalized estimating equations were used to help account for clustering of data within hospitals.

**Results:**

Of 2794 pediatric patients with TBI, 46.6 % were injured in large metropolitan locations, 24.8 % in medium/small metropolitan locations, and 28.6 % in non-metropolitan (rural) locations. Children with rural locations of injury had a greater annualized TBI incidence rate, at 107/100,000 children per year, than those from large metropolitan areas (71/100,000 per year). Compared to children injured in urban locations, those in rural locations had more than twice the crude odds of mortality (odds ratio [OR], 2.5; 95 % CI, 1.6–4.0). This association remained significant (OR, 1.8; 95 % CI, 1.04–3.3) while adjusting for age, gender, race, insurance status, injury severity, and type of TBI (blunt vs. penetrating).

**Conclusion:**

We observed higher rates of TBI and greater proportions of severe injury in rural compared to urban areas in Oregon. Rural children treated in the trauma system for TBI were more likely to die than urban children after controlling for demographic and injury factors associated with urban/rural residence. Further research is needed to examine treatment disparities by urban/rural location. Future work should also identify interventions that can reduce risk of TBI and TBI-related mortality among children, particularly those who live in rural areas.

**Electronic supplementary material:**

The online version of this article (doi:10.1186/s40621-015-0063-2) contains supplementary material, which is available to authorized users.

## Background

Traumatic brain injury (TBI) is a major cause of morbidity and mortality in the United States (US) pediatric population (Hartman et al. 2008; Halldorsson et al. 2007). TBI results in substantial health care costs and many survivors experience permanent disability (Reid et al. 2001; Howard et al. 2005). People who live in urban areas have different physical and socioeconomic environments than those who live rurally; therefore, risk factors for injury and poor outcomes of injury are likely also very different (Kim et al. 2012). These inherent differences between urban and rural location may lead to differences in the epidemiology and outcomes of TBI that have important implications for care and prevention (Gabella et al. 1997). Furthermore, differences in the provision of care can lead to outcomes disparities between children in urban versus rural locations (Grossman et al. 1997; Mullins et al. 2002; Esposito et al. 1995).

Characteristics of TBI tend to be different based on urban versus rural location. Among the few TBI studies that include pediatric populations in the US, injury severity has consistently been reported to be higher in rural compared to urban locations (Reid et al. 2001; Gabella et al. 1997; Chapital et al. 2007). In contrast, studies examining urban/rural differences in pediatric TBI *mortality* have reported mixed results (Gabella et al. 1997; Chapital et al. 2007; Adekoya and Majumder 2004). Compared to children in metropolitan locations, children in rural areas had a higher frequency of motor vehicle related mechanisms of injury, which are known to be associated with increased injury severity compared to other mechanisms, potentially helping explain severity and mortality differences across regions (Reid et al. 2001; Gabella et al. 1997; Chapital et al. 2007; Thurman et al. 1999). Studies also indicate that there may be treatment and care disparities between urban and rural children; pre-hospital, emergency department (ED), and hospital care differences can lead to mortality disparities (Grossman et al. 1997; Mullins et al. 2002; Esposito et al. 1995; Messek et al. 1992; Rutledge et al. 1994; Newgard et al. 2007; Oregon Health Authority, Public Health Division, 2012; Sugerman et al. 2012).

More robust research into urban/rural disparities for children with TBI is needed. Focusing on one state (Oregon), this study is the first to use a multi-level urban/rural geographic classification scheme, developed by the National Center for Health Statistics (NCHS), to examine differences in the incidence, severity, and outcomes (mortality) among pediatric patients treated for TBI. Unlike other commonly used classification schemes, such as the US Census Bureau’s urban and rural designations and the US Office of Management and Budget’s metropolitan and nonmetropolitan designations (which are dichotomous), the NCHS scheme includes six county-based levels: large central metropolitan, large fringe metropolitan, medium metropolitan, small metropolitan, non-metropolitan micropolitan, and non-metropolitan noncore (Ingram and Franco 2012). Thus, the NCHS was specially designed to capture health differences “across the entire urbanization spectrum” and is useful for identifying potential differences in mortality by location due to treatment and care disparities (Ingram and Franco 2012). Additionally, the scheme can aid the development of geography-specific injury prevention and control strategies (Ingram and Franco 2012). We hypothesized that, after accounting for potential confounding factors, mortality among children with TBI would be greater among those in rural locations compared to those in urban locations.

## Methods

### Study population

This retrospective analysis was based on data from the Oregon Trauma Registry maintained by the Oregon Health Authority (OHA) (Oregon Health Authority, Public Health Division 2012 and 2014). The Oregon Trauma Registry includes data from patients who meet at least one of the following criteria: 1) Patient meets pre-hospital triage criteria and is entered into the system by field personnel; 2) Patient requires a surgeon’s evaluation or treatment, or activation of the trauma team in the ED and is entered by ED staff; 3) Patient requires transfer to a trauma center for trauma care and was entered at transfer; or 4) Patient did not receive a trauma team response but retrospectively was entered into the system by either the transfer or receiving facility and had either an Injury Severity Score (ISS) >8; death; a major operative procedure to the head, chest, or abdomen within six hours of hospital arrival, or was admitted to the intensive care unit within 24 h of arrival (Oregon Health Authority, Public Health Division 2014). Retrospective entries do not include non-trauma hospitals, if no transfer was made, or pre-hospital deaths. The study was approved by the OHA Institutional Review Board with ceded oversight from Oregon Health & Science University. The registry was queried from 2009 to 2012 for TBI patients ages 0–19 years. TBI diagnoses were identified using International Classification of Diseases-9^th^ Revision-Clinical Modification (ICD-9-CM) diagnosis codes, as specified in the Barell Injury Diagnosis Matrix (Barell et al. 2002); a TBI code identified in any diagnosis code field was included. We included ICD-9-CM 959.01 indicating a head injury not otherwise specified to prevent under-estimation of TBI as recommended by the US Centers for Disease Control and Prevention (CDC) (Barell et al. 2002; United States Centers for Disease Control and Prevention 1997; Bazarian et al. 2006). Patients who had transfers indicated but without final mortality outcomes available within the Oregon Trauma System database were included in incidence calculations but excluded from subsequent analysis. Demographic data obtained from the registry included age, gender, race, insurance status, zip code/county of injury location, and zip code/county of residence. High agreement between geographic location of injury and location of residence has been previously reported (Gabella et al. 1997). To capture TBI cases occurring in Oregon, regardless of state of residence, we included patients who had an in-state location of injury and excluded patients with an out-of-state location of injury.

### Measures

The primary outcome variable was mortality. Mortality information was obtained directly from the Trauma Registry database and was defined as any death reported in the registry during the study time frame (Oregon Trauma Registry 2002–2012). For those with multiple records due to transfer between hospitals, the record from the final Oregon Trauma Registry hospital was used to assess mortality outcome. County of injury location information was used to assign one of the six urban/rural categories based on the NCHS multilevel classification scheme (Ingram and Franco 2012). To accommodate the relatively small number of outcome events, the scale was collapsed into three categories: large metropolitan (combining large central and large fringe metropolitan), medium/small metropolitan (combining medium and small metropolitan), and non-metropolitan (combining micropolitan and noncore), which was used as the primary independent variable. In the final collapsed categories, large metropolitan included 5 counties and 3 hospitals; small/medium metropolitan included 8 counties and 16 hospitals; and non-metropolitan included 23 counties and 25 hospitals. Patients were assigned 2013 NCHS designations (Ingram and Franco 2012). For the purposes of this analysis, non-metropolitan designations were considered “rural” (Ingram and Franco 2012).

Race categories were categorized as white, non-white, and missing/unknown (Ingram and Franco 2012). Insurance categories were categorized as insured, including those with private insurance, public insurance, car insurance or workers compensation; uninsured, including those who self-paid or received charity; and missing/unknown. Public insurance included Medicare/Medicaid and other government insurance (Howard et al. 2005). The CDC reports that those ages 0–4 and 15–19 years are at the greatest risk for TBI (Thurman et al. 1999; United States Centers for Disease Control and Prevention 1997). Thus, we categorized age as 0–4, 5–14, and 15–19 years.

Injury-related information from the trauma hospital ED to which the patient initially presented was used. Variables included mechanism of injury and intent of injury (based on ICD-9-CM external cause of injury codes, or E-codes), ISS, Glasgow Coma Scale (GCS) score, and TBI-type. Mechanism of injury was categorized as fall, struck by/against, motor vehicle collision (MVC), firearm, and other, specified and unspecified (United States Centers for Disease Control and Prevention 1997). Injury intent was defined as unintentional, suicide attempt, assault, and undetermined/other (United States Centers for Disease Control and Prevention 1997). We categorized ISS as done in past research: mild (≤8), moderate (9–15), and severe (>15) (Stevenson et al. 2001; Copes et al. 1988). However, due to small cell sizes, mild and moderate ISS categories were combined for analysis. The Glasgow Coma Scale measures the level of consciousness in head-injured patients. GCS scores were categorized as mild/moderate (scores = 9–15) versus severe injuries (3–8) (Kim et al. 2012). TBI-type was defined as either blunt or penetrating.

Transfer status to another trauma facility was coded as a dichotomous variable (yes/no). Hospital trauma system level, indicating the level of the trauma hospital to which a patient first presented, was reported as a categorical variable corresponding to the four levels: I, II, III, or IV. Time from initial geographic location of injury to the trauma center was classified as less than 30 min versus 30 min or more.

### Statistical analysis

We used Census data for years 2009-2012 as the denominator to calculate annual TBI incidence rates. We used county Federal Information Processing Standardization (FIPS) codes to determine population totals by urban/rural location (United States Census Bureau 2015). Injury rates for cases with missing location of injury were calculated using Oregon State population totals. We examined differences in demographic and injury variables by urban/rural location of injury using χ^2^ tests. Bivariable logistic regression analysis was performed to investigate the crude association between urban/rural location and mortality. We performed multivariable logistic regression to assess the association between urban/rural location and mortality while controlling for potentially confounding variables. To account for clustering within individual hospitals, we used generalized estimating equations (GEEs) in all logistic regression analyses (Hardin and Hilbe 2007). An a priori causal model and directed acyclic graphs were used to determine the most parsimonious sets of available covariates to include in analyses (Greenland et al. 1999). The first multivariable model adjusted for age, sex, race, and insurance status. The second model added injury severity and TBI-type as covariates. We explored multiple indicators of injury severity including ISS, GCS score, systolic blood pressure (SBP), diastolic blood pressure, pulse rate, and respiratory rate. We determined that ISS would serve as the best proxy of severity for final multivariable analysis due to the low percentage of missing values (1.4 %) compared to other potential variables and its common use as a severity indicator in the TBI literature (e.g. Reid et al. 2001; Gabella et al. 1997). We computed adjusted odds ratios (ORs) and 95 % confidence intervals (CIs) to estimate risk of TBI-related mortality for children in non-metropolitan and small/medium metropolitan areas compared with large metropolitan areas.

All analyses were performed using Stata/SE 13.1 (StataCorp 2013).

## Results

### TBI incidence

There were 3169 children aged 0–19 years treated for TBI in the Oregon Trauma System from 2009 to 2012. The average incidence rate of pediatric TBI in Oregon over the four year period was 87 per 100,000 children per year. The incidence rates of TBI among those with non-metropolitan locations of injury were greater than those of the large and medium/small metropolitan areas for all years examined (Table 1).

**Table 1:** Annual TBI incidence rates^a^ for Oregon children ≤19 years of age, by urban/rural location

	Year				
Injury location (Total Population)	2009	2010	2011	2012	Average
Large metropolitan	75 (460,120)	73 (456,364)	71 (455,593)	68 (455,576)	71 (456,913)
Small/medium metropolitan	66 (309,021)	64 (307,284)	59 (305,168)	48 (301,299)	59 (305693)
Non-metropolitan	111 (206,886)	108 (208,535)	110 (205,271)	101 (202,415)	107 (205,777)
Missing location^b^	17 (976,027)	10 (972,183)	8 (966,032)	13 (959,250)	12 (968,373)
Combined incidence	96 (976,027)	87 (972,183)	83 (966,032)	80 (959,250)	87 (968,373)

*TBI* traumatic brain injury

^a^ Per 100,000 children per year

^b^ Rates calculated using Oregon population totals for denominators

### Patient characteristics

After excluding patients without a reported geographic location of injury (n = 247; 8 %) and patients for whom final disposition could not be assessed (n = 128; 4 %), the study population for assessing associations between injury location and mortality included 2794 patients. (Excluded children were slightly more likely to be older, have injuries classified as severe, have a missing GCS score, and have an unknown/missing insurance status. Excluded children were also less likely to present to either a Level I or II trauma hospital.) There were 1303 patients injured in a large metropolitan location (47 %), 692 in a medium/small metropolitan location (25 %), and 799 in a non-metropolitan location (29 %). Overall, patients were primarily white (72 %) and male (67 %). The most frequent mechanisms of injury were falls (34 %) and MVCs (33 %).

The characteristics of children and their injuries varied by injury location (Table 2). Children sustaining TBI in rural locations were more likely to be white and between the ages of 15–19 compared to children in large-metropolitan locations. Furthermore, rural children were more likely to be uninsured or classified as having unknown/missing insurance. MVCs were the most common mechanism of injury among rural children (37 %), while falls were the most common mechanism among urban children (39 %).

**Table 2:** Characteristics of children admitted to Oregon trauma hospitals for TBI, 2009–2012, by urban/rural location

	Large metropolitan (*n* = 1303)		Small/medium metropolitan (*n* = 692)		Non-metropolitan (*n* = 799)		Total (*n* = 2794)	
	*n*	(%)	*n*	(%)	*n*	(%)	*n*	*p*
Injury Severity Score								<0.0001
≤15 (mild/moderate)	984	(76)	477	(69)	507	(63)	1968	
>15 (severe)	316	(24)	198	(29)	272	(34)	786	
Missing	3	(0)	17	(2)	20	(3)	40	
Glasgow Coma Scale score								<0.0001
9–15 (mild/moderate)	1161	(89)	496	(72)	626	(78)	2283	
3–8 (severe)	42	(3)	34	(5)	29	(4)	105	
Missing	100	(8)	162	(23)	144	(18)	406	
Gender								0.97
Male	879	(67)	463	(67)	537	(67)	1879	
Female	424	(33)	229	(33)	262	(33)	915	
Age (years)								0.02
0–4	325	(25)	163	(24)	149	(19)	637	
5–14	380	(29)	203	(29)	240	(30)	823	
15–19	598	(46)	326	(47)	410	(51)	1334	
Race								<0.0001
White	891	(68)	538	(78)	578	(72)	2007	
Non-white	348	(27)	136	(20)	91	(11)	575	
Missing/unknown	64	(5)	18	(3)	130	(16)	212	
Insurance status								<0.0001
Insured	1197	(92)	579	(84)	611	(76)	2387	
Uninsured	84	(7)	52	(8)	62	(8)	198	
Other/unknown/missing	22	(2)	61	(9)	126	(16)	209	
Transfer status								<0.0001
Yes	23	(2)	129	(19)	195	(24)	347	
No	1280	(98)	563	(81)	604	(76)	2447	
Mechanism of injury								<0.0001
Fall	509	(39)	204	(29)	227	(28)	940	
Motor vehicle collision	367	(28)	250	(36)	298	(37)	915	
Struck by/against	188	(14)	80	(12)	75	(9)	343	
Firearm	7	(0)	3	(0)	3	(<1)	13	
Other, specified and unspecified	232	(18)	155	(22)	196	(25)	583	
Hospital Level^a^								<0.0001
I	1251	(96)	76	(11)	149	(19)	1476	
II	5	(0)	401	(58)	211	(26)	617	
III	1	(0)	129	(19)	249	(31)	379	
IV	46	(4)	86	(12)	190	(24)	322	

*TBI* traumatic brain injury

^a^ Hospital level refers to the level of trauma hospital to which a patient initially presented

Severity of injuries was also different by location (Table 2). Of the children with a rural location of injury, 34 % sustained severe traumatic injuries compared to 24 % of children from large metropolitan locations. Children from rural locations had a higher percentage of missing GCS scores (18 %) compared to children from large metropolitan areas (8 %). Of the children with a recorded GCS score, there was no difference in the proportion of severe TBIs by location; 4 % in rural locations were categorized as severe compared to 3 % in large metropolitan locations. Of the children injured in rural locations, 20 % experienced transportation times greater than 30 min versus 15 % for large metropolitan locations. Approximately 75 % of all children were transported to trauma hospitals by ambulance; however, children who were injured in rural locations were twice as likely to be transported by helicopter (15 % of the total rural cases) as children in large metropolitan areas. A slightly higher percentage of injuries in rural locations were unintentional compared to those in large metropolitan areas, although the difference was statistically insignificant (95 % vs. 92 %).

### Mortality

Of the 76 total deaths reported in the Trauma Registry database during the study time frame, 82 % occurred within three days, and 88 % within 5 days, of the initial injury date. One patient was identified as dead on arrival. Less than 7 % of deaths (*n* = 5) occurred in excess of 10 days from the date of injury. We observed a significant difference between geographic location of injury and mortality outcome, with crude mortality of 1.8 % for large-metropolitan location of injury, 2.3 % for small/medium metropolitan location of injury, and 4.5 % for non-metropolitan location of injury (Table 3). Children injured in non-metropolitan (rural) locations were 2.5 times more likely to die compared to those in large metropolitan locations (OR, 2.5; 95 % CI, 1.6–4.0) (Table 4). In contrast, there was no statistically significant difference in odds of mortality for patients injured in small/medium metropolitan locations compared to those in large metropolitan locations (OR, 1.3; 95 % CI, 0.6–2.8). When controlling for potential confounders (age, race, gender, insurance), the estimated adjusted odds of mortality among children injured in a non-metropolitan location, compared to those in large metropolitan locations, was 2.4 (95 % CI, 1.4–3.9) (Additional file 1: Table S5). This association was further attenuated by additional adjustment of ISS and injury type (OR 1.8; 95 % CI, 1.04–3.3) (Additional file 1: Table S6). When adjusting for SBP in addition to demographic variables, the association was only slightly attenuated (OR 2.3; 95 % CI, 1.3–4.2) and was still statistically significant (Additional file 1: Table S7). The difference in mortality between patients injured in small/medium versus large metropolitan locations remained statistically non-significant after adjusting for potential confounders in all models. Although we were unable to test the interaction between transfer status and ISS in multivariable models due, in part, to small cell sizes, we did observe a significant association at the bivariable level between transfers and mortality among children with severe injuries (OR 0.3; 95 % CI, 0.2–0.6). There was no observed association between transfer status and mortality among children with mild/moderate injury severity.

**Table 3:** Characteristics of children admitted to Oregon trauma hospitals for TBI, 2009–2012, by mortality outcome

	Lived (*n* = 2718)		Died (*n* = 76)		Total (*n* = 2794)	
	*n*	(%)	*n*	(%)	*n*	*p*
Urban/Rural location						0.001
Large metropolitan	1279	(98)	24	(2)	1303	
Medium/small metropolitan	676	(98)	16	(2)	692	
Non-metropolitan	763	(95)	36	(5)	799	
Injury Severity Score						<0.0001
≤15 (mild/moderate)	1960	(100)	8	(0)	1968	
>15 (severe)	720	(92)	66	(8)	786	
Missing	38	(95)	2	(5)	40	
Glasgow Coma Scale score						<0.0001
9–15 (mild/moderate)	2280	(100)	3	(0)	2283	
3–8 (severe)	82	(78)	23	(22)	105	
Missing	356	(88)	50	(12)	406	
Gender						0.83
Male	1827	(97)	52	(3)	1879	
Female	891	(97)	24	(3)	915	
Age (years)						0.013
0–4	612	(96)	25	(4)	637	
5–14	811	(99)	12	(1)	823	
15–19	1295	(97)	39	(3)	1334	
Race						0.022
White	1956	(97)	51	(3)	2007	
Non-white	562	(98)	13	(2)	575	
Missing/unknown	200	(94)	12	(6)	212	
Insurance status						0.01
Insured	2332	(97)	55	(3)	2387	
Uninsured	185	(96)	13	(4)	198	
Other/unknown/missing	201	(97)	8	(3)	209	
Transfer status						0.209
Yes	334	(96)	13	(4)	347	
No	2384	(97)	63	(3)	2447	
Mechanism of injury						<0.0001
Fall	928	(99)	12	(1)	940	
Motor vehicle collision	879	(96)	36	(4)	915	
Struck by/against	341	(99)	2	(1)	343	
Firearm	6	(46)	7	(54)	13	
Other, specified and unspecified	564	(97)	19	(3)	583	
Hospital Level^a^						0.053
I	1447	(98)	29	(2)	1476	
II	596	(97)	21	(3)	617	
III	363	(96)	16	(4)	379	
IV	312	(97)	10	(3)	322	
Injury type						<0.0001
Blunt	2695	(98)	69	(2)	2764	
Penetrating	13	(65)	7	(35)	20	
Missing	10	(100)	0	(0)	10	

*TBI* traumatic brain injury

^a^ Hospital level refers to the level of trauma hospital to which a patient initially presented

**Table 4:** Odds of mortality by urban/rural location among Oregon children with TBI

Urban/Rural classification			Multivariable Associations			
	Bivariable Association		Model 1^a^		Model 2^b^	
	OR	95 % CI	OR	95 % CI	OR	95 % CI
Large metropolitan	1.0	Ref	1.0	Ref	1.0	Ref
Small/medium metropolitan	1.3	(0.6, 2.8)	1.2	(0.6, 2.6)	1.1	(0.5, 2.5)
Non-metropolitan	2.5	(1.6, 4.0)	2.4	(1.4, 3.9)	1.8	(1.04, 3.3)

*TBI* traumatic brain injury

^a^ Model 1 includes age, gender, race, and insurance

^b^ Model 2 includes age, gender, race, insurance, ISS, and injury type (blunt versus penetrating)

### Post Hoc analyses

To further assess potential causes for the differences indicated in logistic regression analyses, we conducted post hoc analyses examining ED treatment and transfer variables. Of children with a rural location of injury, 95 % were indicated as receiving no treatment in the ED to which they initially presented, compared to 84 % of children with a large metropolitan location of injury. (Children were more often discharged [30 % of all children], transferred [12 %], or sent to other units/departments [51 %].) Of the 205 children with large metropolitan locations of injury who were reported as receiving some type of ED treatment, 17 % had intubation listed as their initial treatment; none of those with rural locations of injury, and only 5 % of those with small/medium metropolitan locations of injury, had an initial treatment of intubation. Among children who initially presented to a Level III or IV trauma hospital and had an ISS categorized as severe (ISS >15), only 69 % were transferred to Level I or II hospitals. Of those injured in small/medium metropolitan locations, 8 % who were not transferred died, compared with 4 % who were transferred. Of those injured in rural locations, 37 % who were not transferred died, compared with 7 % of those who were transferred.

## Discussion

This is the first study to use the NCHS Urban/Rural Classification Scheme, which is specially designed to capture health differences, to examine potential urban/rural disparities in TBI-related incidence, severity, and mortality among children using trauma system care. We found that there was a greater incidence rate of trauma system care for TBI among children in rural areas compared to large metropolitan areas. Our results also indicated that rural children treated in the trauma system for TBI were more likely to die than urban children while controlling for injury severity and other factors likely associated with urban/rural residence. A greater proportion of children in non-metropolitan areas sustained severe overall trauma, as indicated by the ISS, when compared to those with TBI from large metropolitan locations. Frequencies of primary mechanism tended to differ between urban and rural locations, although intent of injury did not significantly differ between locations.

Our findings suggest that systematic differences in care and/or treatment could be contributing to increased mortality in children sustaining TBIs in rural locations. Currently, longer transport times are thought to be associated with the increased risk of injury mortality in rural areas (Adekoya and Majumder 2004; Tiesman et al. 2007). Studies have reported decreased chances of survival with transportation times from injury scene to initial care over 30 min (Grossman et al. 1997). In our study, a larger proportion of children injured in rural locations experienced transportation times greater than 30 min; however, this difference was relatively small (20 % versus 15 % for large metropolitan locations) and likely does not explain all of the increased mortality observed in rural children. Treatment received during pre-hospital transportation may also contribute to mortality disparities. Rural locations are more likely to have volunteer emergency medical technicians (EMTs) with less experience due to low retention of workers (Grossman et al. 1997). Additionally, rural areas have fewer trained advanced life support responders (such as paramedics) compared to large metropolitan locations. Basic life support trained responders do not have the training necessary to intubate patients or provide intravenous medications or fluids (Grossman et al. 1997). Given that rural responding EMTs are less likely to be able to provide such care, we would expect these treatments to occur upon arrival to the ED. However, analysis of treatment received in the trauma hospital of initial presentation indicated that 95 % of children with rural locations of injury received no ED treatment upon arrival, compared with 84 % of children with large metropolitan locations of injury. Furthermore, of the approximately 5 % of rural children who were listed as receiving some type of ED treatment, none had an initial treatment of intubation. Future research should focus on identifying differences in time to care, modes of transportation, and disparities in both pre-hospital and ED treatment by urban versus rural location and assessing how such disparities contribute to mortality outcomes.

Our findings are consistent with previous reports of differences in incidence and outcome of TBI based on location. Gabella et al. (1997) reported rates of 98 per 100,000 for most urban groups and 172 per 100,000 for most rural groups in Colorado. Although our study suggested a similar trend in relative incidence, the incidence rates for our study are lower due to our focus on the pediatric population. Our overall average incidence of 87 per 100,000 children is similar to that reported by Reid et al. (2001) in their study of pediatric TBI in Minnesota (74 per 100,000 children) (Reid et al. 2001). Gabella et al. also reported increased TBI-related mortality in rural locations compared to urban locations. Chapital et al. (2007) found no statistically significant differences in TBI mortality rates for all ages between urban and rural locations, although findings did suggest a higher rate in the rural population. We believe that differences across studies may be accounted for, at least in part, by differences in defining urban versus rural locations. Further research focusing on the pediatric population should use consensus-driven urban/rural classification schemes such as the NCHS Classification Scheme, which is designed to identify health differences.

Similar to previous studies, falls and MVCs accounted for the highest proportions of TBI mechanisms in our population (Reid et al. 2001; Gabella et al. 1997). Our findings are consistent with previous reports that MVCs are more commonly the primary mechanism of injury among those in non-metropolitan locations (Reid et al. 2001; Ingram and Franco et al. 2012). MVCs have been shown to be significantly associated with greater TBI severity while falls were not (Reid et al. 2001). This may partially explain the observed increased injury severity for children with rural locations of TBI. Research into rural location-specific injury prevention strategies for MVCs and severity-reduction strategies for injuries sustained in MVCs should be conducted to guide development of prevention plans targeted towards rural children.

This retrospective analysis has several limitations. Due to the relatively small number of outcome events (mortality) in conjunction with a high number of missing variables for GCS scores, we used ISS to measure overall severity of children’s injuries. ISS does not directly measure TBI severity; however, severe head injuries are accounted for in the ISS score. Consequently, patients with severe levels of TBI should have a high ISS translating to a severe injury classification for our study. Despite this, ISS may not adequately control for severity given it does not account for multiple severe injuries in the same body region. We therefore included TBI type (blunt versus penetrating) in addition to ISS as an indicator of severity in our multivariable analyses. Other physiological measures, such as SBP, would potentially provide additional information on injury severity; however, adjustment for SBP in our multivariable analysis only slightly attenuated the significantly elevated risk of mortality amongst those with rural locations of injury compared to large metropolitan locations of injury. The distribution of SBP entries in this population indicated that SBP of a patient may be entered as zero following death, rather than entered as the SBP upon initial presentation. Such reporting bias may artificially strengthen associations between SBP and mortality. Also related to low cell counts, we were unable to assess effect modification of the association between urban/rural location of injury and mortality by either ISS or transfer status in regression analyses. We had wanted to assess a larger time period, but registry data quality issues and reporting differences between years prevented utilization of data prior to 2009. It is unknown how missing data for severity indicators, or for outcome (*n* = 128; 4 %), may have influenced results. Future studies should be conducted in larger populations with more complete reporting to help disentangle associations between urban/rural location, TBI and overall injury severity, transfer status, and risk of mortality.

Another limitation is the potential for selection bias if there was systematic underreporting or overreporting by region. Cases that did not have an Oregon location of injury or where the geographic location of injury (*n* = 247; 8 %) was missing were excluded from location-specific incidence calculations and multivariable analyses. We found that those we excluded were more likely to be older, have injuries classified as severe, have a missing GCS score, and have an unknown/missing insurance status. They were also less likely to present to either a Level I or II trauma hospital. These characteristics are consistent with characteristics of children in our analysis population with non-metropolitan and small/medium metropolitan locations of injury. Since children who had injuries classified as severe, who had missing GCS scores, and who presented to Level III or IV trauma centers had higher crude mortality in the analysis population, we could be missing deaths at a higher rate in the non-metropolitan and small/medium metropolitan locations in the excluded population. This would lead to falsely attenuated odds of mortality for non-metropolitan and small/medium metropolitan locations. If location was less likely to be reported for rural areas, this could lead to an under sampling of rural TBI cases causing an underreporting primarily of rural TBI. The registry does not capture pre-hospital mortality cases or patients admitted to non-trauma system hospitals. In our population, transportation times were longer for rural locations of injury which could lead to higher rates of death prior to presentation at the ED. Rural patients may also be admitted to non-trauma hospitals at a higher rate and die prior to transfer. Data from rural hospitals across Oregon and Washington have shown that over 10 % of patients in non-trauma centers die in non-trauma center EDs awaiting transfer to trauma centers (Mullins et al. 2002). Thus, mortality cases in rural areas could be excluded at a greater rate than urban mortality cases, potentially resulting in falsely reduced odds of mortality for children in rural areas. Future studies should use methods to enumerate children with TBI who may have died prior to receipt of care in the Trauma System.

## Conclusion

Our study found mortality disparities as well as differences in the epidemiology of TBI by urban versus rural locations. Children in rural areas had higher TBI incidence rates as well as greater odds of dying from their injuries. Our results suggest that there was a greater burden of TBI mortality among rural children, after adjusting for severity of injury. Such differences may be due to disparities in treatment and care, and further examination of specific treatment and care disparities are needed amongst pediatric patients who sustain TBIs. Additionally, differences in mechanisms and severity of injury suggest that location-specific TBI prevention programs may be warranted.

## Additional file

Additional file 1:
**Supplemental content.** **Table S5** Odds ratios for multivariable regression model 1 covariates. **Table S6.** Odds ratios for multivariable regression model 2 covariates. **Table S7.** Odds ratios for multivariable regression model 1 after addition of SBP. (DOC 53 kb)

## Abbreviations

CDC: Centers for Disease Control and Prevention
CI: Confidence interval
E-code: External cause of injury code
ED: Emergency department
GCS: Glasgow Coma Scale
GEE: Generalized estimating equations
ICD-9-CM: International Classification of Diseases, 9th Revision, Clinical Modification
ISS: Injury Severity Score
LOS: Length of stay
MVC: Motor vehicle collision
NCHS: National Center for Health Statistics
OHA: Oregon Health Authority
OR: Odds ratio
TBI: Traumatic brain injury
US: United States
