# Supplementary material for: Urban/Rural disparities in Oregon pediatric traumatic brain injury
Source: Inj Epidemiol. 2015 Dec 11;2(1):32. doi: 10.1186/s40621-015-0063-2 (PMC4676786; doi:10.1186/s40621-015-0063-2)
Supplement: Additional file 1: — Supplemental content. Table S5 Odds ratios for multivariable regression model 1 covariates. Table S6. Odds ratios for multivariable regression model 2 covariates. Table S7. Odds ratios for multivariable regression model 1 after addition of SBP. (DOC 53 kb) [file 40621_2015_63_MOESM1_ESM.doc]

**Additional file 1**

**Table S5** Odds ratios for multivariable regression model 1covariates

| **Model 1 Covariates** | **OR** | **95% CI** |
| --- | --- | --- |
| Age 0-4 years | 1.0 | Ref |
| Age 5-14 years | 0.3 | (0.1, 0.7) |
| Age 15-19 years | 0.6 | (0.4, 0.97) |
| Male | 1.0 | Ref |
| Female | 0.9 | (0.6, 1.4) |
| White race | 1.0 | Ref |
| Non-white race | 0.9 | (0.6, 1.5) |
| Unknown race | 1.8 | (0.9, 3.6) |
| Insured | 1.0 | Ref |
| Uninsured | 2.8 | (1.5, 5.0) |
| Unknown/Missing insurance status | 1.4 | (0.6, 3.2) |

Note. TBI = traumatic brain injury

**Table S6** Odds ratios for multivariable regression model 2 covariates

| **Model 2 Covariates** | **OR** | **95% CI** |
| --- | --- | --- |
| Age 0-4 years | 1.0 | Ref |
| Age 5-14 years | 0.3 | (0.2, 0.8) |
| Age 15-19 years | 0.7 | (0.4, 1.1) |
| Male | 1.0 | Ref |
| Female | 1.1 | (0.8, 1.5) |
| White race | 1.0 | Ref |
| Non-white race | 0.8 | (0.5, 1.3) |
| Unknown race | 1.7 | (0.6, 4.4) |
| Insured | 1.0 | Ref |
| Uninsured | 3.4 | (1.8, 6.4) |
| Unknown/Missing insurance status | 1.3 | (0.5, 3.6) |
| ISS < 15 (mild/moderate) | 1.0 | Ref |
| ISS > 15 (severe) | 21.9 | (6.8, 70.3) |
| Blunt TBI | 1.0 | Ref |
| Penetrating TBI | 10.3 | (5.9, 18.2) |

Note. TBI = traumatic brain injury

**Table S7** Odds ratios for multivariable regression model 1 after addition of SBP

| **Model 1 Variables** | **OR** | **95% CI** |
| --- | --- | --- |
| Large metropolitan injury location | 1.0 | Ref |
| Small/medium metropolitan injury location | 1.7 | (0.6, 4.4) |
| Non-metropolitan injury location | 2.3 | (1.3, 4.2) |
| Age 0-4 years | 1.0 | Ref |
| Age 5-14 years | 0.5 | (0.2, 1.0) |
| Age 15-19 years | 1.5 | (0.8, 2.6) |
| Male | 1.0 | Ref |
| Female | 0.6 | (0.4, 1.0) |
| White race | 1.0 | Ref |
| Non-white race | 0.9 | (0.6, 1.3) |
| Unknown race | 1.5 | (0.9, 2.4) |
| Insured | 1.0 | Ref |
| Uninsured | 2.5 | (1.4, 4.3) |
| Unknown/Missing insurance status | 1.3 | (0.6, 3.1) |
| SBP | 1.0 | (0.9, 1.0) |

Note. TBI = traumatic brain injury; SBP = systolic blood pressure
